# High expression of caspase‐8 as a predictive factor of poor prognosis in patients with esophageal cancer

**DOI:** 10.1002/cam4.5496

**Published:** 2022-12-19

**Authors:** Jian Chai, Yongqiang Lei, Xindong Xiang, Jing Ye, Hang Zhao, Lili Yi

**Affiliations:** ^1^ Joint Laboratory for Translational Medicine Research, Beijing Institute of Genomics Chinese Academy of Sciences & Liaocheng People's Hospital Liaocheng China; ^2^ The Key Laboratory of Molecular Pharmacology Liaocheng People's Hospital Liaocheng China; ^3^ Department of Pathology Liaocheng People's Hospital Liaocheng China; ^4^ Department of Thoracic Surgery Liaocheng People's Hospital Liaocheng China

**Keywords:** CASP8, esophageal cancer, immune infiltration, prognosis

## Abstract

**Background:**

Esophageal carcinoma (ESCA) is considered to be one of the most common gastrointestinal cancers. Caspase‐8 (CASP8) is a key protein of cross‐talk signaling in a variety of cancers. However, the role of CASP8 expression in the prognosis of patients with ESCA has remained unexplored. Hence, it is needed to explore the clinical significance of CASP8 expression in ESCA.

**Methods:**

The expression and prognosis of CASP8 were investigated in ESCA using the UALCAN, GEDS, TIMER, and OncoLnc databases. The CASP8 genetic variations in ESCA were assessed using the cBioPortal database. The correlation between CASP8 expression and tumor immune invasion and immune cell surface indicators was examined using the TIMER and TISIDTISIDB datasets. Meanwhile, the abundance of the immunological cells in the tumor and healthy tissues was assessed by the CIBERSORT method. Next, information on the co‐expressed genes of the differentially expressed genes (DEGs) in ESCA‐tumor and ESCA‐healthy tissues was obtained using the cBioPortal, UALCAN, and Coexpedia databases. Subsequently, the PPI network was constructed and the GO and KEGG pathways were analyzed using the SIRING database. Finally, CASP8 mRNA and protein expression in the ESCA tissues and matched adjacent healthy tissues were analyzed using qRT‐PCR, immune‐blotting, and immunohistochemistry. Additionally, the relationship between clinicopathological features and CASP8 expression was assessed.

**Results:**

ESCA tissues had higher levels of CASP8 mRNA and protein expression compared to healthy tissues. patients with an elevated level of CASP8 expression had poor overall survival (OS). CASP8 expression was significantly correlated with the degree of differentiation (P = 0.004) and lymph node metastasis (P = 0.044). There were diverse patterns of association between immunological cell surface biomarkers and CASP8 expression.

**Conclusion:**

ESCA showed significant levels of CASP8 expression which may serve as a prognostic biomarker correlated to immune infiltrates in ESCA.

## Graphical abstract

CASP8 mRNA and protein expression in ESCA tissues were high than normal tissues by qPCR, WB, and IHC. Patients with CASP8 high expression have poor overall survival. Correlation of CASP8 expression with immune cells was present. CASP8 may serve as a prognostic biomarker associated with immune infiltrates in ESCA.

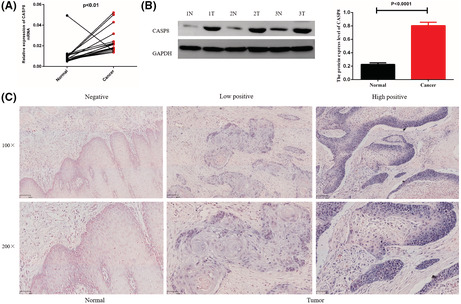

## 1 INTRODUCTION

Esophageal carcinoma (ESCA) is the most common type of cancer with a poor prognosis. Approximately, esophageal squamous cell carcinoma (ESCC) is considered to be the sixth leading cause of cancer‐associated deaths worldwide.^1^ Multiple recent studies indicated that the incidence of esophageal cancer is continuously increasing.^2^ Nowadays, ESCC is the most prevalent pathological type of ESCA in the Chinese population, accounting for greater than 85% of ESCA cases.^3^, ^4^ The poor prognosis of ESCA patients is mostly related to late diagnosis and rapid metastasis. In this view, it is needed to identify candidate prognostic biomarkers to enhance the prognosis and therapeutic efficacy of ESCA patients.

Caspases are aspartate‐specific cysteine proteases that serve as key regulators of apoptotic cell death occurring via the intrinsic and extrinsic pathways.^5^ The aberrant expression or function of apoptotic signaling pathways is a common hallmark of cancer.^6^, ^7^ Caspase‐8 is an important mediator of signal transduction from death receptors to the pro‐apoptotic machinery within cells. In the absence of caspase‐8, cells can undergo necroptotic cell death via an alternate signaling cascade mediated by MLKL (mixed‐lineage kinase domain‐like) and RIPK1/RIPK3.^8^ While knocking out caspase‐8 in mice is lethal even at an embryonic stage. However, animals with a double knockout of caspase‐8/RIPK3 or caspase‐8/MLKL develop normally.^9^, ^10^ Caspase‐8 can thus serve as an inhibitor of necroptosis by blocking RIPK3 activation. Caspase‐8 has also been correlated with the regulation of oncogenic progression, tissue homeostasis, and recovery following injury.^11^, ^12^, ^13^, ^14^, ^15^ However, the precise functional significance of caspase‐8 in tumor progression is still a matter of considerable debate, as it has been observed to be alternately upregulated and downregulated in cancer cells.^15^ Low caspase‐8 levels are also linked to a worse prognosis for ovarian cancer patients and increase the risk of metastatic growth for neuroblastoma and neuroendocrine lung tumors.^16^, ^17^, ^18^, ^19^, ^20^ In pancreatic and breast cancer, conversely, caspase‐8 upregulation has been linked to enhanced tumor cell migration.^21^, ^22^ There is also some data linking it to a lower chance of survival for those with hepatocellular carcinoma. Nuclear localization of caspase‐8 has been shown in a variety of malignancies, suggesting it may have tumorigenic roles apart from inducing cell death in an oncogenic scenario.^23^, ^24^

Few studies have examined the association between caspase‐8 and ESCA development to date. Therefore, the current study assessed the correlations between caspase‐8 expression levels and ESCA diagnostic and prognostic factors to determine the key role of caspase in this type of cancer. Furthermore, It was also investigated whether immune cell infiltration and caspase‐8 are related.

## 2 MATERIALS AND METHODS

### 2.1 Tissue samples

Patients with ESCA who were admitted to the Liaocheng People's hospital from March 2019 to February 2021 and had complete follow‐up data were enrolled in this study (52 males, 10 females; 23 patients aged 60 years, 39 patients aged >60 years). There were 62 tumor tissues and 16 normal tissues from 62 individuals with ESCA, cancer tissues (all SCC as proven by histology) and mucosal tissues >6 cm away from the tumor margin. Tumor and matched normal tissues were all analyzed by immunohistochemistry (IHC). And 16 sample pairs were analyzed by Quantitative Real‐Time Polymerase Chain Reaction (RT‐qPCR). And three sample pairs were chosen for western blot analysis. The inclusion criteria were as follows: having undergone surgery without radiation, chemotherapy, or any other specific treatment. There were a total of 24 cases with low differentiation and 38 cases with high differentiation. In addition, TNM staging was used to categorize 62 individuals as I + II = 33 and III + IV = 29. In accordance with the degree of lymph node metastasis, the lymph nodes could be categorized as metastatic (*n* = 33) or non‐metastatic (*n* = 29). We divided them into positive (*n* = 27) or negative (*n* = 35) cases based on whether vessel carcinoma embolus appeared in pathology. Moreover, the patients were classified as either positive (*n* = 29) or negative (*n* = 33) depending on whether perineural invasion is present or not. The approval of the underlined study was provided by Liaocheng People's Hospital's Ethical Committee and the informed consent form was signed by each patient.

### 2.2 Expression of CASP8 in ESCA

The UALCAN database (http://ualcan.path.uab.edu)^25^ provided information on the CASP8 expression level and clinicopathological features in ESCA that was accessible. Meanwhile, the GEDS (http://bioinfo.life.hust.edu.cn/web/GEDS/)^26^ and the tumor immune estimation resource (TIMER) database (https://cistrome.shinyapps.io/timer/)^27^ were used to assess the expression level of CASP8 in ESCA.

### 2.3 Survival analysis using the OnconLc database

The Kaplan plot for CASP8 in ESCA was obtained from the OncoLnc database (http://www.oncolnc.org).^28^

### 2.4 RNA‐sequencing data

RNA‐seq data from 848 ESCA cases were obtained from the Cancer Genome Atlas (TCGA) (https://cancergenome.nih.gov/)^29^ and Genotype‐Tissue Expression (GTEx) (https://gtexportal.org/home/)^30^ databases. For further analysis, the level 3 HTSeq‐FPKM (fragments‐per‐kilobase per million) formats of the retrieved data were changed to TPM (transcripts per million) formats. The area under the curve (AUC) of CASP8 was analyzed to determine whether CASP8 may be used as a biomarker to distinguish between the surrounding tissues and the tumor.

### 2.5 TIMER database analysis

Using the TIMER database, the correlation between CASP8 expression and the relative abundance of immunological cells that infiltrate the body (T cells, B cells, CD8^+^, CD4^+^, T cells, macrophages, neutrophils, and dendritic cells) in ESCA patients were examined. It was hypothesized that tumor integrity was a crucial factor determining immune infiltration in tumor samples when using genomic approaches.

### 2.6 TISIDB database analysis

TISIDB is an online site for tumor‐immune system interactions that combines numerous diverse data sources in Oncoimmunology, including PubMed and TCGA literature mining results.^31^ Herein, the TISIDB database was used to examine CASP8 expression levels in tumor‐infiltrating lymphocytes (TILs) of human cancers.

### 2.7 Cell‐type identification by estimating relative subsets of RNA transcripts (CIBERSORT)

CIBERSORT is a method for assessing the relative proportions of distinct cell subsets in tissues that are based on the input matrix of a gene expression file.^32^ This study used the GEPIA2021 database and the CIBERSORT approach to estimate the number of immune cells in normal tissues and tumor tissues.

### 2.8 Gene alterations of CASP8 in ESCA using cBioPortal

cBioPortal (http://www.cbioportal.org)^33^ was used to examine CASP8 gene mutations for ESCA. ESCA (TCGA, Nature 2017) was selected for further analysis. OncoPrint was generated in cBioPortal (TCGA preliminary) to directly represent all forms of variations in the CASP8 amplification, mRNA upregulation, deep deletion, and mRNA downregulation in patients with ESCA. Additionally, the potential effect of CASP8 mutations on the survival of ESCA patients was evaluated using Kaplan–Meier survival curves in the cBioPortal.

### 2.9 Identification of differentially expressed genes and screening of co‐expressed genes

The differentially expressed genes (DEGs) were evaluated between the ESCA and healthy tissues with |Log_2_FC (fold change)|>1 and *p* < 0.01. Subsequently, co‐expressed genes of DEGs in ESCA‐tumor and ESCA‐normal tissues were collected from cBioportal, UALCAN, and Coexpedia (https://www.coexpedia.org).^34^

### 2.10 PPI network construction, GO function, and KEGG pathway analysis

The STRING version‐11.0 database,^35^ which is based on selected databases, PubMed abstracts, experimental/biochemical data, and other bioinformatics resources, contained both known and predicted PPI networks. CASP8 was used as a search input to identify possible proteins that interact with CASP8. The default scoring threshold for interaction was 0.4, and a subnetwork containing genes that may interact with CASP8 was retrieved. A network made up of the interacting and driving genes for CASP8 was constructed. Next, all of the selected genes were subjected to KEGG pathway analysis and gene ontology (GO) enrichment using the STRING database.

### 2.11 Quantitative real‐time polymerase chain reaction

The total RNA extraction of ESCA and adjacent tissues was carried out using the FastPure Cell/Tissue Total RNA Isolation Kit as per the provided protocol (A260/280 = 1.8–2.0) of the manufacturer. Using the Superscript III RT kit, cDNA was synthesized, followed by qPCR analyses using a RT‐qPCR SYBR kit. Experiments were repeated three times. The following CASP8 primer sequences were used as forward and reverse primers: forward, 5′‐CAAACTTCACAGCATTAGGGAC‐3′; reverse, 5′‐ATGTTACTGTGGTCCATGAGTT‐3′. GAPDH was used as an internal control. Finally, the relative quantification values of CASP8 were calculated using the 2^^‐ΔΔCT^ formula.

### 2.12 Immunohistochemistry

For each 10 min, paraffinized tissue sections were dewaxed (three times) using xylene (Yantai Fast Eastern Fine Chemical CO., LTD.). The sections were serially rehydrated from 100% ethanol, followed by 95%, 90%, 80%, 70%, 60% ethanol, and distilled water. Antigen retrieval was carried out for 10 min in an EDTA buffer at pH 9.0. After a 30 min incubation at 37°C in 5% BSA, they were treated with a mouse anti‐CASP8 monoclonal antibody (sc‐56070; Santa Cruz Biotechnology) at a concentration of 1:100 for 24 h at 4°C. The sections were three times washed for 5 min using PBS (pH 7.2) as a washing buffer. After 30 min at 37°C and three washes in PBS buffer, the biotin‐conjugated secondary anti‐mouse antibody (BOSTER detection system, SA1051) was used to detect the binding of the anti‐CASP8 antibody. The sections were then exposed to SABC‐AP (SA1051, BOSTER) for 30 min at 37°C before being washed four times for 5 min in 0.01 M TBS (pH 9.0–9.5). Following that, the samples were processed for chromogen development for 30 min using BCIP/NBT. Nuclear Fast Red (SA1051, BOSTER) was used as a counterstain on the portions for 5 min. To determine the staining intensity and staining positivity rate for the CASP8 expression, three pathologists employed a double‐blind procedure to randomly choose five high‐power visual fields. score for staining intensity: 0 (negative), 1 (positive), and 2 (strong positive). Positive score: 0, 1 (1%–25%), 2 (26%–50%), 3 (51%–75%), 4 (76%–100%). The total score of <4 was considered a low CASP8 expression, while ≥4 indicated a high CASP8 expression.

### 2.13 Western blotting

Fresh tissues (30 mg) were harvested and the proteins were extracted using the RIPA buffer (P0013B, Beyotime Biotechnology) with 1 mM PMSF (ST506, Beyotime Biotechnology) and 1X protease inhibitor cocktail (P1010, Beyotime Biotechnology). The extracted proteins were quantified by BCA (P0010, Beyotime Biotechnology). The primary antibodies listed below were treated with the PVDF membrane at 4°C overnight: anti‐human CASP8 (1:1000 dilution; sc‐56070, Santa Cruz Biotechnology) and anti‐GAPDH (1:1000 dilution; 5174, CST). The membrane was washed with 0.5% TBST thricely for 5 min. Next, the membrane was exposed to secondary antibodies conjugated with HRP (1:5000 dilutions; ZB2305, CST) for 2 hrs. at 25°C. After chemiluminescence, the images were statistically analyzed under the ImageJ software.

### 2.14 Statistical Analysis

Box plots were used to identify the gene expression distributions, and the Wilcoxon test was used to assess the statistical significance of differential expression in UALCAN, TIMER, and GEDS. In the Kaplan–Meier Plotter curve, the log‐rank test was used to obtain the HR and log‐rank *p*‐value. R statistical software (version 3.6.3) was used to analyze ROC. The ESCA Correlation module generates expression scatterplots between CASP8 together with the estimated statistical significance and Spearman's rho value. The correlations between different immune checkpoint receptors on TILs and CASP8 were evaluated by Spearman correlation analysis. The two‐tailed unpaired Student's *t* test or one‐way ANOVA for multiple comparisons was used to analyze CASP8 expression in tumor and matched normal tissues. The relationship between clinicopathological features and CASP8 expression was analyzed using the chi‐squared test. The data were analyzed to use the Graph Pad Prism 6.0 and SPSS software. In the absence of special circumstances, *p* < 0.05 was considered statistically significant (*p < 0.05, **p < 0.01, ***p < 0.001, ****p < 0.0001).

## 3 RESULTS

### 3.1 Expression of CASP8 in ESCA

**FIGURE 1:**
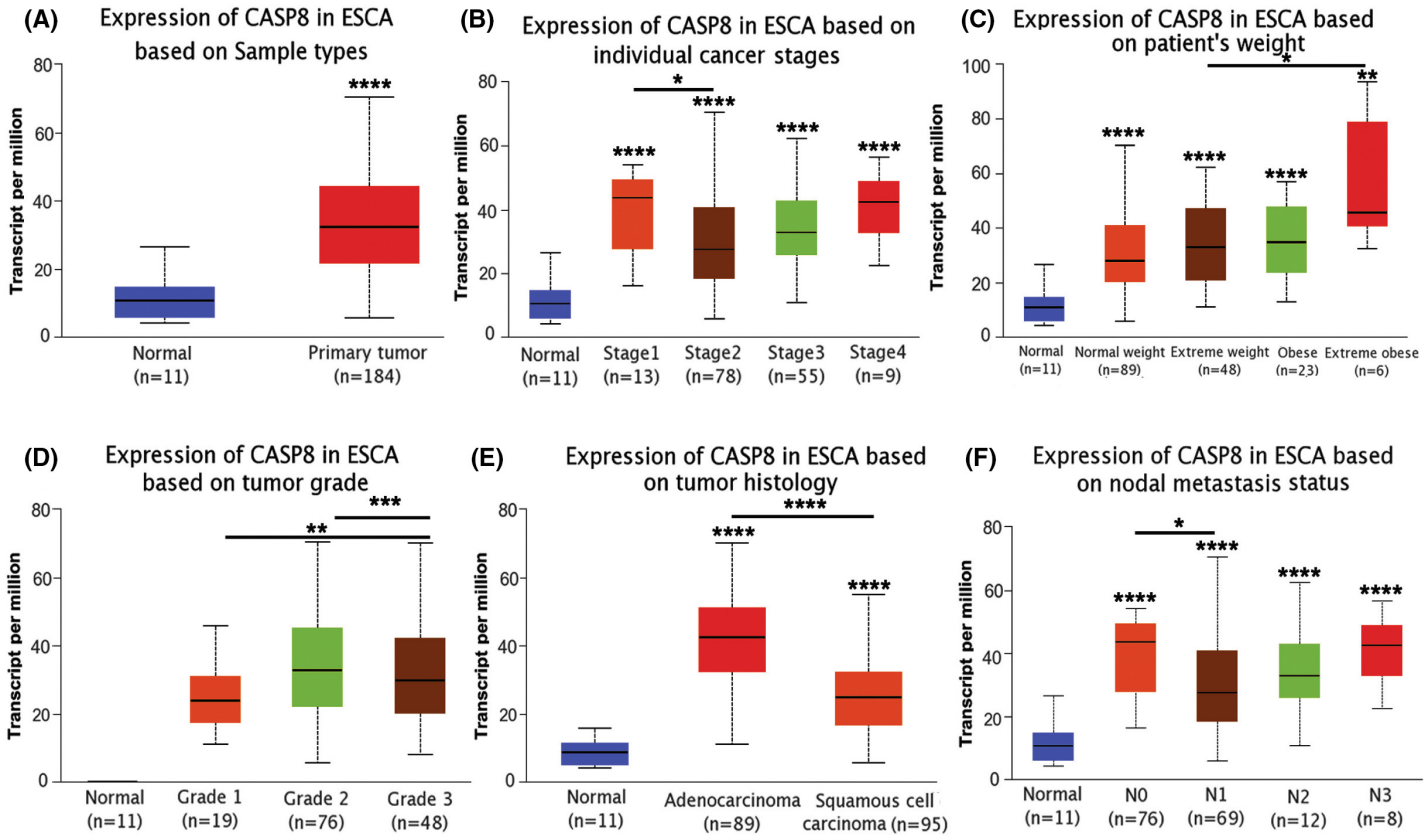
UALCAN‐based expression of CASP8 mRNA in ESCA. Expression of CASP8 in ESCA based on different (A) sample types, (B) cancer stages, (C) patient's weight, (D) tumor grade, (E) tumor histology, and (F) lymph node status.

**FIGURE 2:**
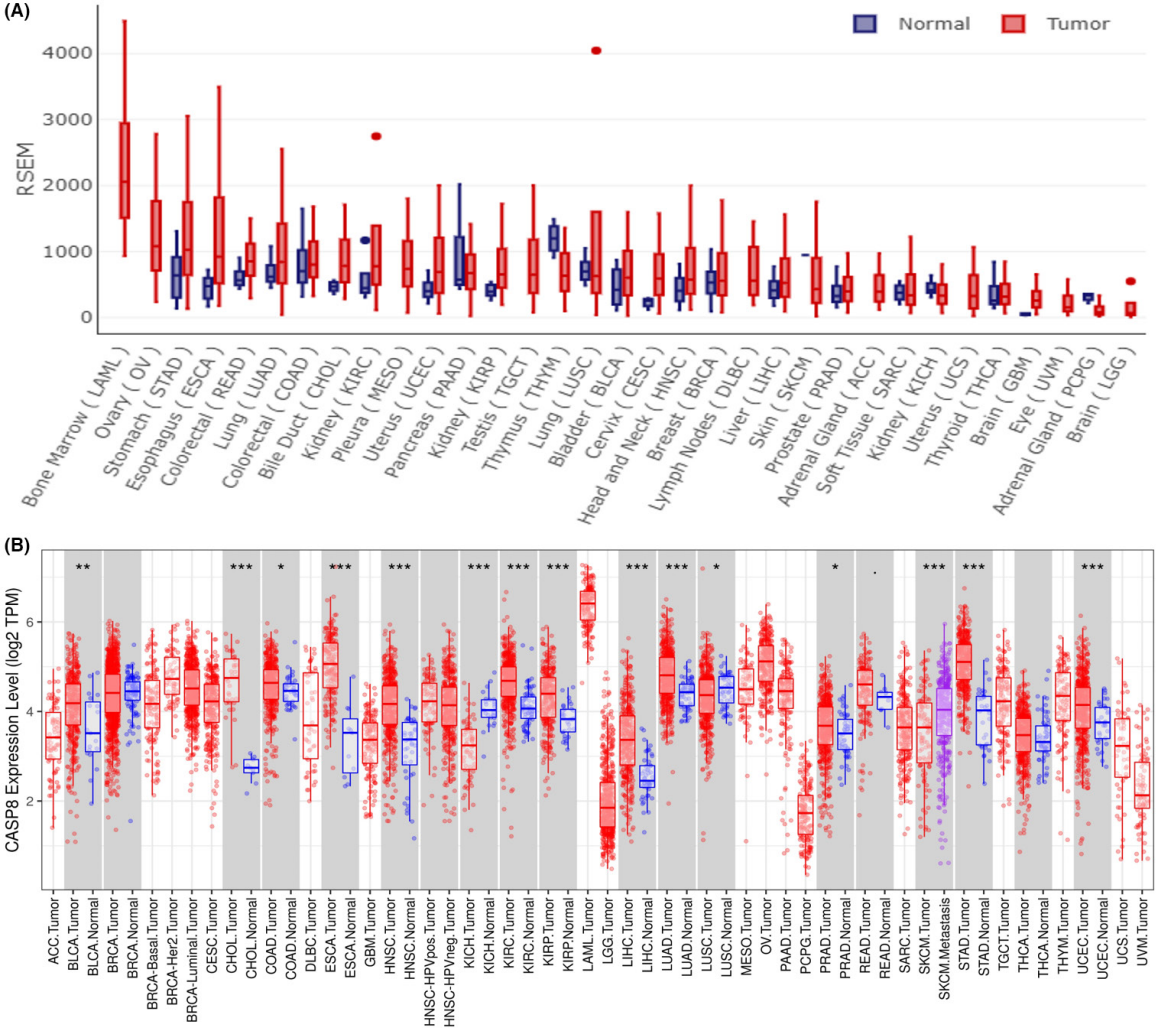
The transcription levels of CASP8 in human cancers. The mRNA expression of CASP8 between tumor and normal tissues was evaluated by the (A) GEDS and (B) TIMER databases.

The obtained results of this study demonstrated that ESCA tissues had an elevated level of CASP8 expression compared with healthy tissue's CASP8 expression level (Figure 1A, and Figure 2). Based on specific cancer stages, when the expression of CASP8 was analyzed in ESCA we observed that the levels of expression in the four cancer stages were elevated when compared to healthy cells, with the maximum expression level occurring in stage 1 (Figure 1B). Moreover, the CASP8 expression level was found to be positively correlated with the body weight of the patients (Figure 1C). The levels of expression in all three grades, but especially in grade 2, were higher than in normal cells (Figure 1D). ESCA includes adenocarcinoma and squamous cell adenocarcinoma. The CASP8 expression level was found to be the highest in adenocarcinoma (Figure 1E). Additionally, the four stages of lymph node involvement exhibited greater CASP8 mRNA expression than healthy tissues (Figure 1F).

### 3.2 Survival of patients with ESCA based on CASP8 expression

**FIGURE 3:**
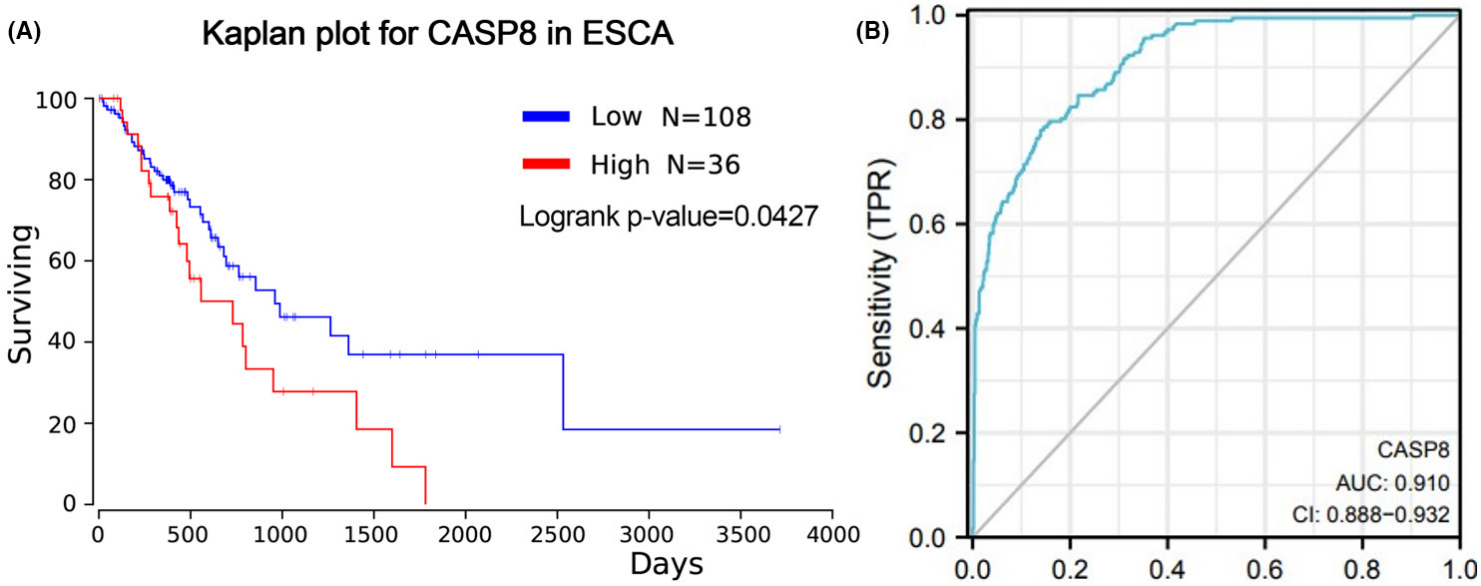
Overall survival rate and ROC for CASP8 in ESCA. (A) The overall survival rate for CASP8 by Kaplan plot. (B) The ROC curve showed the efficacy of the CASP8 expression level in differentiating BC tumors from normal tissue. The X‐axis shows the number of false positives, and the Y‐axis shows the number of actual positives.

Subsequently, an overall survival analysis based on CASP8 expression was performed using the Kaplan–Meier curve. According to the obtained results, patients having an elevated expression of CASP8 displayed poor overall survival (Figure 3A). The efficiency of the CASP8 expression level in differentiating the ESCA tissues from the healthy tissues was also examined using the ROC curve. The AUC of CASP8 was found to be 0.91, which suggests that CASP8 may work as a candidate biomarker in distinguishing ESCA from healthy tissues (Figure 3B).

### 3.3 Correlation between CASP8 expression and immune cell infiltration

**FIGURE 4:**
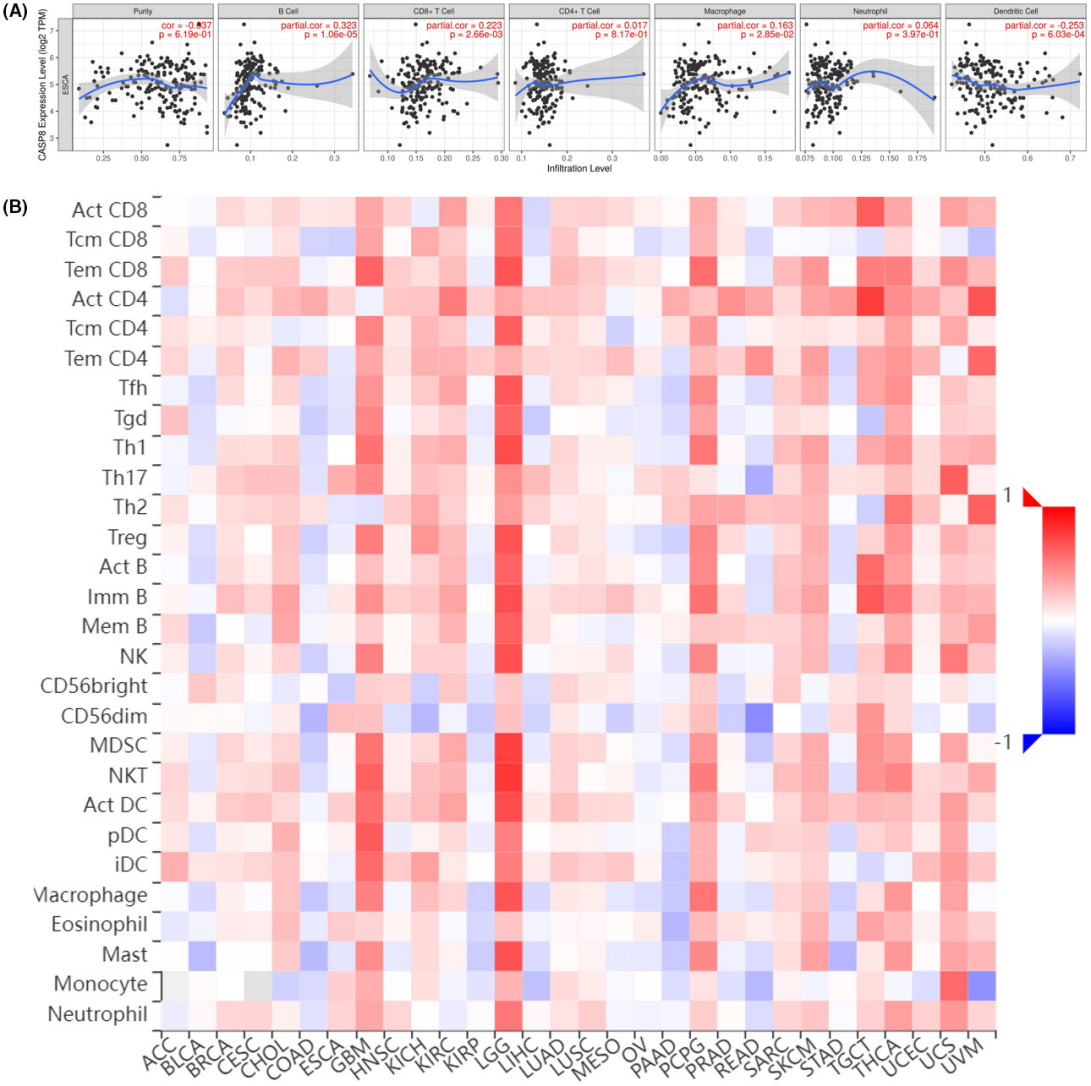
Correlation between the expression of CASP8 and immune cells. Correlation of CASP8 expression with immune cells using the TIMER database. (A) Using the TISIDB database, a correlation analysis was performed between the expression of CASP8 and the number of tumor‐infiltrating lymphocytes (TILs) in human cancers.

Immune cell infiltration is closely correlated with the progression and development of a variety of tumors. Herein, TIMER was used to figure out if there was a link between CASP8 expression and immune infiltration. This was done by calculating the CASP8 expression coefficient index with immune infiltration in ESCA. We assessed six different immunological cell types that infiltrate tissues: CD4^+^ T cells, B cells, macrophages, dendritic cells, CD8^+^ T cells, and neutrophils. In ESCA, CASP8 expression showed a positive correlation with B cells (*r* = 0.323, *p* = 1.06 e‐05), CD8^+^ T cells (*r* = 0.223, *p* = 2.66 e‐03), and macrophages (*r* = 0.163, *p* = 2.85 e‐02), but it showed a negative correlation with dendritic cells (*r* = −0.253, *p* = 6.03 e‐04). However, CASP8 expression was not associated with tumor purity (*r* = −0.037, *p* = 6.19 e‐01), CD4^+^ T cells (*r* = 0.017, *p* = 8.17 e‐01), and neutrophils (*r* = −0.064, *p* = 3.97 e‐01) (Figure 4A). Additionally, CASP8 expression levels in TILs of human malignancies were analyzed using the TISIDB database. CASP8 expression was positively associated with Th 17 cells, CD56dim cells, and Eosinophil cells in ESCA and negatively associated with Tcm_CD8 cells and CD56bright cells (Figure 4B).

### 3.4 CASP8 expression and immunological cell markers' correlation

The presence of cellular markers characterizes immunological cells, such as M1/M2 macrophages, tumor‐associated macrophages, monocytes, CD8^+^ T cells, B cells, NK cells, neutrophils, and dendritic cells. Different functional T cells were evaluated in this study, as shown in Table 1. After adjusting for tumor purity in TIMER, a substantial correlation was observed between CASP8 expression levels and 27 of the 44 immune cell biomarkers.

**TABLE 1:** Correlations between CASP8 and gene markers of immune cells

Cell type	Gene marker	None		Purity	
		Cor	*p*	Cor	*p*
B cell	CD19	0.253	5.14 e‐04***	0.268	2.74 e‐04***
	CD20	0.216	3.10 e‐03**	0.228	2.08 e‐03**
	CD38	−0.05	5.01 e‐01	−0.064	3.93 e‐01
CD8^+^ T cell	CD8A	0.170	2.10 e‐02*	0.182	1.43 e‐02
	CD8B	0.196	7.51 e‐03**	0.204	5.93 e‐03**
Tfh cell	CXCR5	0.171	2.03 e‐02*	0.174	1.92 e‐02*
	ICOS	0.248	6.89 e‐04***	0.276	1.77 e‐04***
	BCL‐6	−0.281	1.12 e‐04***	−0.289	8.19 e‐05***
Th1 cell	IL12RB2	−0.182	1.31 e‐02*	−0.185	1.31 e‐02*
	WSX‐1	−0.117	1.14 e‐01	−0.124	9.62 e‐02
	T‐BET	0.187	1.08 e‐02*	0.201	6.82 e‐03**
Th2 cell	CCR3	0.171	2.01 e‐02*	0.163	2.93 e‐02*
	STAT6	0.438	6.99 e‐10***	0.431	1.60 e‐09***
	GATA3	0.005	9.44 e‐01	−0.003	9.66 e‐01
Th9 cell	TGFBR2	0.430	1.49 e‐09***	0.420	4.31 e‐09***
	IRF4	0.301	3.41 e‐05	0.315	1.67 e‐05***
	PU.1	−0.025	7.36 e‐01	−0.027	7.17 e‐01
Th17 cell	IL‐21R	0.164	2.61 e‐02*	0.179	1.62 e‐02*
	IL‐23R	0.461	3.91 e‐11***	0.465	4.59 e‐11***
	STAT3	0.235	1.31 e‐03**	0.218	3.24 e‐03**
Th22 cell	CCR10	0.146	4.76 e‐02*	0.147	4.90 e‐02*
	AHR	0.188	1.05 e‐02*	0.176	1.79 e‐02*
Treg cell	FOXP3	0.168	2.22 e‐02*	0.188	1.17 e‐02*
	CCR8	0.240	9.77 e‐04***	0.249	7.64 e‐04***
	CD25	0.099	1.79 e‐01	0.099	1.88 e‐01
T cell exhaustion	PD‐1	0.188	1.05 e‐02*	0.202	6.52 e‐03**
	CTLA4	0.197	7.40 e‐03**	0.212	4.34 e‐03**
Macrophage	CD68	0.155	3.58 e‐02*	0.170	2.29 e‐02*
	CD11b	0.075	3.09 e‐01	0.077	3.05 e‐01
M1	NOS2	0.366	3.64 e‐07	0.364	5.03 e‐07
	ROS1	−0.088	2.35 e‐01	−0.089	2.35 e‐01
M2	ARG1	−0.110	1.37 e‐01	−0.104	1.66 e‐01
	MRC1	0.082	2.69 e‐01	0.090	2.29 e‐01
TAM	HLA‐G	0.101	1.70 e‐01	0.115	1.24 e‐01
	CD80	0.117	1.11 e‐01	0.130	8.12 e‐02
Monocyte	CD14	−0.010	8.88 e‐01	−0.007	9.25 e‐01
	CD16	0.018	8.10 e‐01	0.029	7.04 e‐01
NK	XCL1	−0.166	2.43 e‐02*	−0.154	3.95 e‐02*
	KIR3DL1	0.155	3.50 e‐02*	0.149	4.62 e‐02*
	CD7	0.228	1.84 e‐03**	0.235	1.51 e‐03**
Neutrophil	CD15	0.547	0	0.548	1.62 e‐15***
	MPO	0.108	1.42 e‐01	0.125	9.47 e‐02
DC	CD1C	−0.043	5.64 e‐01	−0.045	5.51 e‐01
	CD141	−0.334	3.81 e‐06***	−0.356	9.45 e‐07***

### 3.5 The immune cells' abundance in ESCA

**FIGURE 5:**
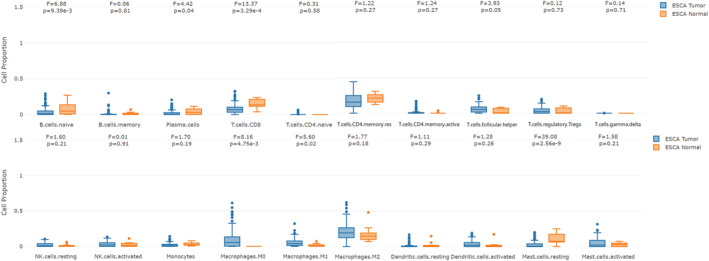
The abundance of immune cells in tumors and normal tissues. The abundance of the immune cells in the tumor and normal tissues was evaluated by the bioinformatics tool CIBERSORT in the GEPIA2021 database.

To analyze the characteristics of TIME (tumor immune microenvironment) in ESCA, the CIBERSORT method was used to evaluate the abundance of 20 immune cells in the tumor and healthy tissues using the GEPIA database. According to the obtained results, the abundance of B cells naïve, plasma cells, T cells CD8 and mast cells resting in normal tissues were higher than those in tumor tissues. Furthermore, the expression levels of Macrophages M0, Macrophages M1 and T cells follicular helper in tumor tissues were higher compared to those in healthy tissues (Figure 5).

### 3.6 cBioPortal gene variations in CASP8 of ESCA tissue

**FIGURE 6:**
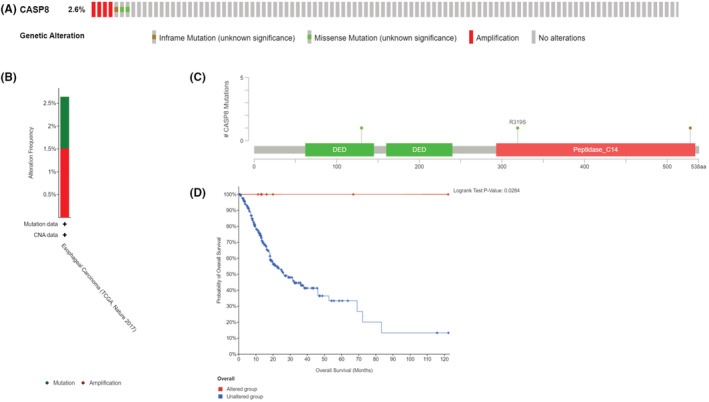
Gene alterations in *CASP8* in ESCA. (A) Mutations in *CASP8* in ESCA are shown. (B) Frequency of gene alterations in *CASP8* in ESCA. (C) Types of mutation in ESCA. (D) Kaplan–Meier survival curves analyzed the correlation of gene alterations in *CASP8* with the overall survival rate.

Gene variations in CASP8 were present in 2.6% of the sequenced cases in the OncoPrint‐schematic of cBioPortal (Figure 6A). Furthermore, 1.5% CASP8 amplification and 1% mutation occurred in esophageal adenocarcinoma (Figure 6B). All mutation types of CASP8 in ESCA are described in Figure 6C. Two missense mutations and one in‐frame mutation were detected in CASP8. Additionally, the relationship between CASP8 gene modifications and patient survival was evaluated. The obtained data revealed a significant correlation (log‐rank *p*‐value = 0.0284) between the overall survival of patients with ESCA and gene alterations in CASP8 (Figure 6D).

### 3.7 Identification of DEGs in ESCA and PPI network construction

**FIGURE 7:**
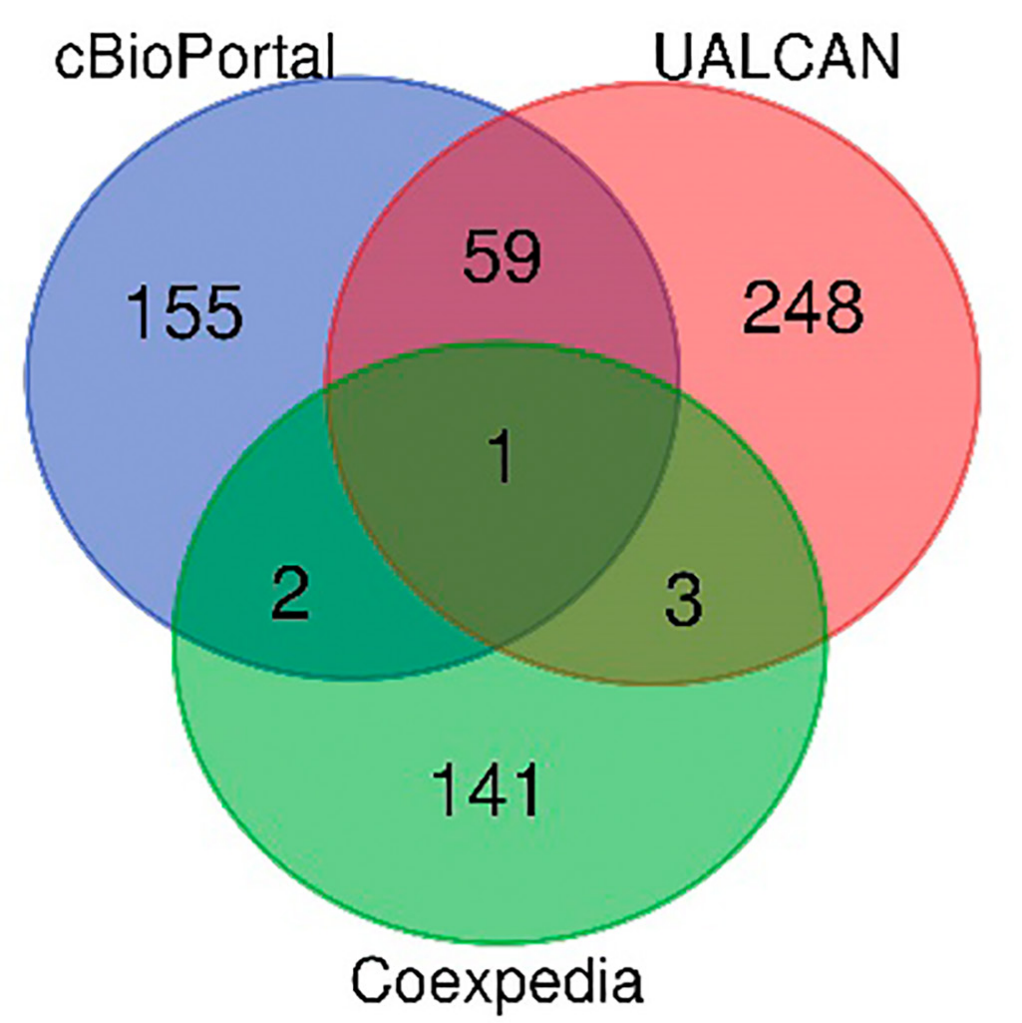
Venn diagram of CASP8 co‐expression genes

As shown in the Venn diagram (Figure 7), 217 co‐expressed genes were obtained from cBioPortal, 311 from UALCAN, and 147 genes from Coexpedia; the only overlapping gene in the three datasets was *LY75* (lymphocyte antigen 75).

### 3.8 PPI network construction, GO function, and KEGG pathway analysis

**FIGURE 8:**
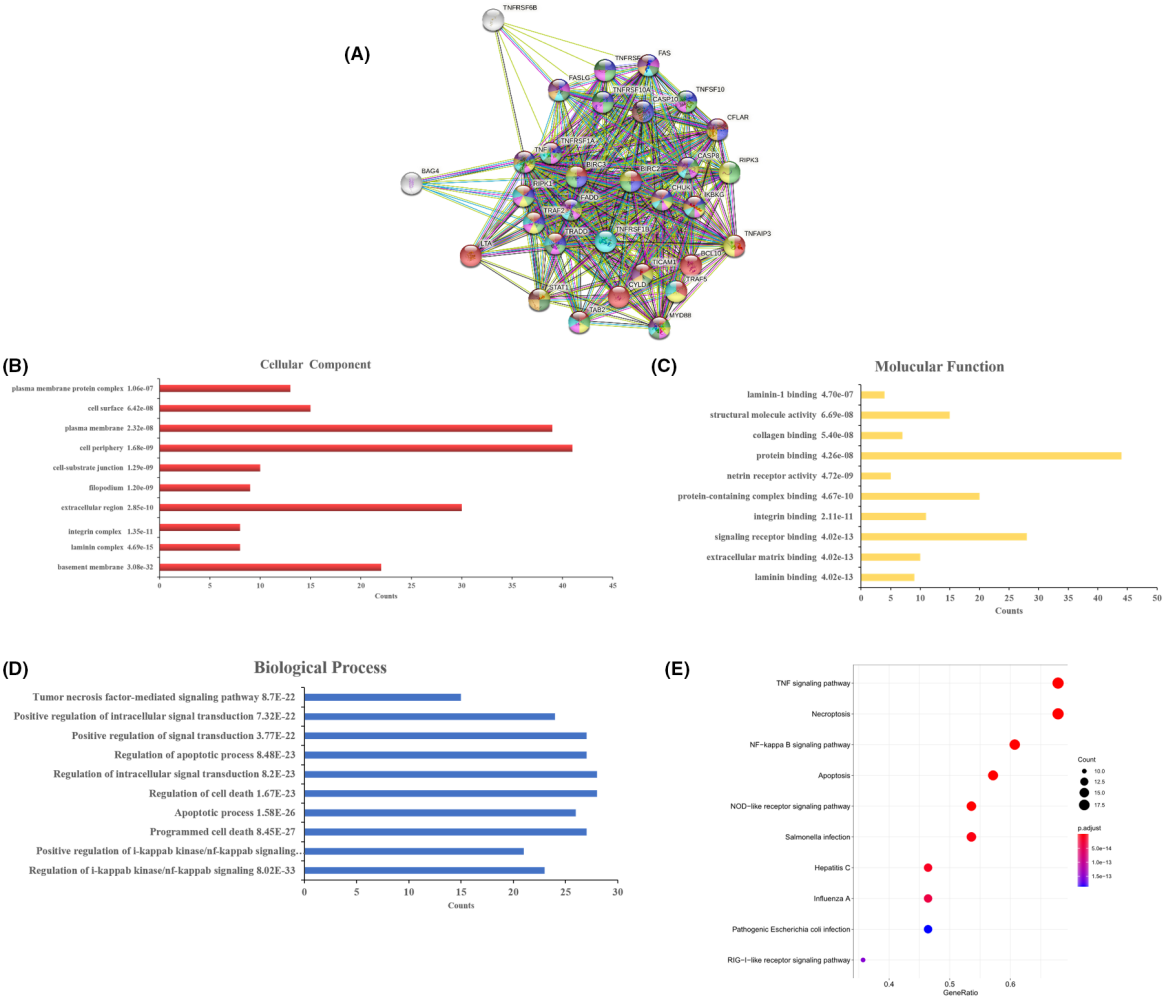
Bioinformatics‐based analysis of CASP8 molecular functions and regulatory pathways. (A) Interaction network of CASP8 based on the STRING database. (B) Cellular component. (C) Molecular function. (D) GO biological process. (E) Pathway enrichment based on KEGG.

The putative molecular function and regulatory pathway of CASP8 were analyzed to reveal a possible mechanism through which CASP8 regulates the biological activities of ESCA. CASP8‐interacting genes in the STRING database were initially searched (Figure 8A). These specified genes underwent GO analysis to uncover the biological processes (BP) (Figure 8D), molecular functions (MF) (Figure 8C), and cellular components (CC) in which interacting genes of CASP8 were involved. According to CC, proteins that were differentially expressed were primarily membrane components. According to BP, proteins with differential expression were mostly engaged in the regulation of programmed cell death, cellular apoptosis, and signal transduction. According to MF, signaling receptor binding was the primary function of differentially expressed proteins. KEGG pathway analysis was carried out to determine the molecular cascades in which the CASP8 interacted genes were active. In Figure 8E, the top 10 pathway enrichments are shown. These included the TNF signaling pathways, NOD‐like receptor signaling pathways, necroptosis, influenza A, NF‐kappa B signaling pathways, apoptosis, *Escherichia coli* infection, *Salmonella* infection, hepatitis C, pathogenic, and RIG‐like receptor signaling cascade.

### 3.9 CASP8 elevated expression in ESCA tissues

**FIGURE 9:**
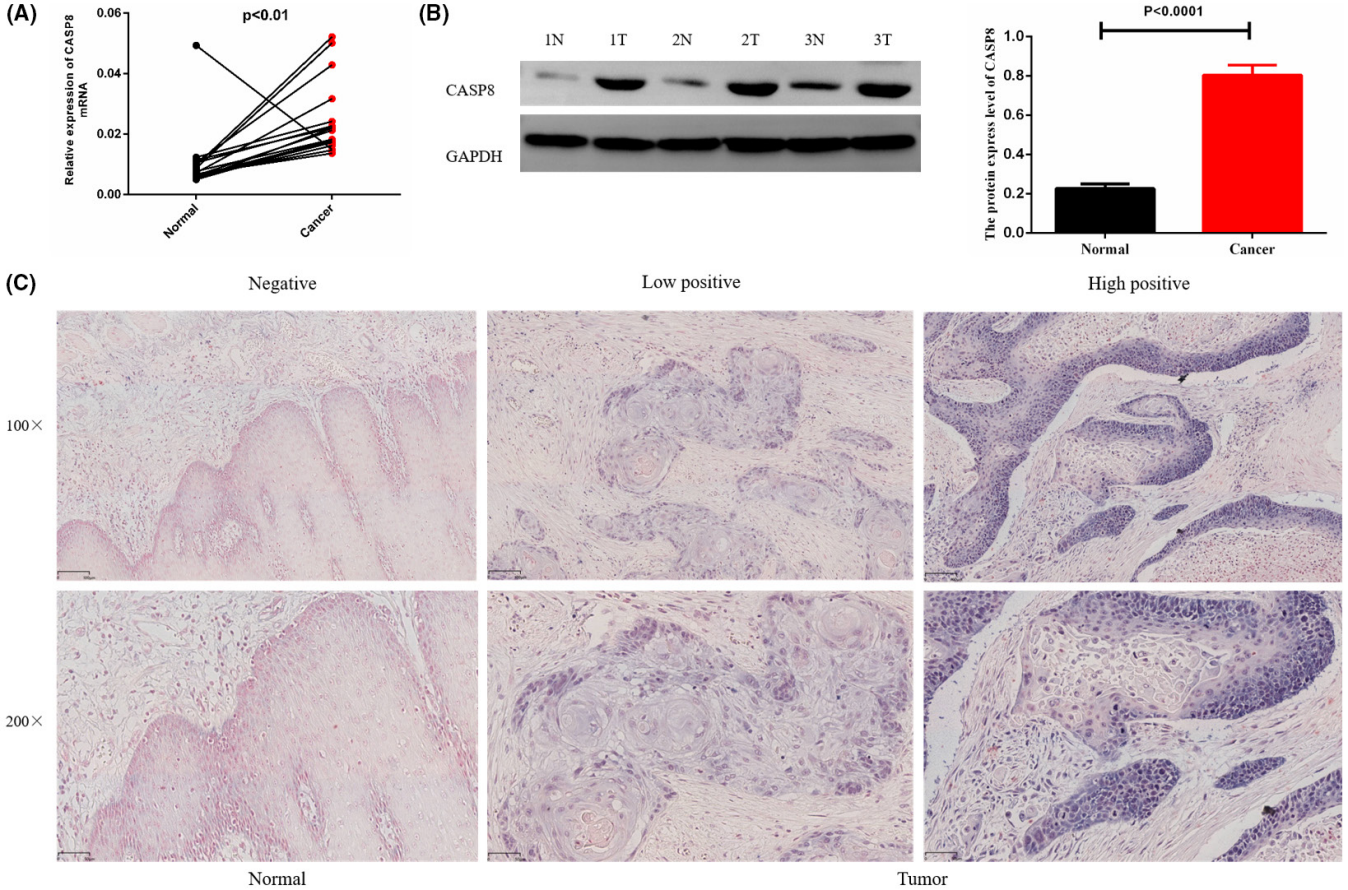
CASP8 expression in ESCA tumor and normal tissues. (A) RT‐qPCR analysis of the CASP8 expression in tumor and surrounding healthy tissue. (B) Western blotting of CASP8 in tumor tissues, and normal tissue. (C) IHC analysis revealed differential CASP8 expressions. Herein, the 200x magnification was the lower, and 100x was the upper.

To comprehend the function of CASP8 expression in ESCA patients, 18 pairs of ESCA tumor tissues and surrounding healthy tissues were examined by RT‐qPCR. The expression of CASP8 mRNA in tumor tissues was observed to be higher than in the adjacent healthy tissues (Figure 9A). Subsequently, CASP8 protein expression was evaluated using western blotting (WB) in three random pairs of cancerous and healthy tissues. As presented in Figure 9B, CASP8 protein expression was higher in ESCA tissues than in the surrounding healthy tissues. Moreover, CASP8 expression was evaluated in 62 ESCA tumor tissues and 18 healthy tissues by IHC. As per the IHC scores, 18/62 (29.1%) ESCA tumor tissues demonstrated weak CASP8 staining and 44/62 (70.9%) showed strong staining. On the other hand, in the adjacent healthy tissues, 14/18 (77.8%) exhibited weak staining and 4/18 (22.2%) showed strong staining (Figure 9C).

### 3.10 Correlation of clinicopathological features with CASP8 protein expression

Herein, the association between CASP8 expression and clinicopathological characteristics was further explored. Table 2 summarizes the association between CASP8 expression and clinical and pathological characteristics. Correlation and regression analysis indicated that CASP8 expression was considerably associated with differentiation (*p* = 0.004) and lymph node metastasis (*p* = 0.044). However, no significant associations were identified with age, sex, TNM stage, vessel carcinoma embolus, and nerve invasion. These data demonstrate that increased CASP8 expression may promote tumor growth of ESCA.

**TABLE 2:** Clinical and pathological aspects of esophageal carcinomas with different levels of CASP8 protein expression

Clinical pathology	Samples	CASP8		*χ* ^2^	*p*
		low	high		
Tissue types					
Cancer tissues	62	18	44	13.811231	0.00020
Adjacent tissues	18	14	4		
Sex					
Male	52	14	38	0.696115	0.404092
Female	10	4	6		
Age (years)					
≤60	23	10	13	3.703467	0.054300
>60	39	8	31		
Differentiated degree					
Low differentiated	24	2	22	8.142788	0.004323
High and middle differentiated	38	16	22		
TNM stage					
I + II	33	10	23	0.055297	0.814090
III + IV	29	8	21		
Lymph node metastasis					
Yes	33	6	27	4.031443	0.044660
No	29	12	17		
Vessel carcinoma embolus					
Positive	27	8	19	0.008284	0.927480
Negative	35	10	25		
Perineral invasion					
Positive	29	8	21	0.055297	0.814090
Negative	33	10	23		

## 4 DISCUSSION

Esophageal cancer is one of the highly prevalent gastrointestinal cancer in China. This severe type of cancer is characterized by late diagnosis and high mortality. Despite the advances in surgical and non‐surgical treatment in the past decades, the prognosis remains to be poor. The main reason for the poor prognosis of esophageal cancer is the unavailability of specific biomarkers for early diagnosis and the unavailability of effective treatment. According to the obtained results of the present study, CASP8 expression was significantly elevated in the ECSA tumor tissues when compared with the adjacent healthy tissues through RT‐qPCR and WB analyses. Furthermore, database analysis revealed a correlation between elevated CASP8 mRNA levels and a bad prognosis in ESCA patients. Using IHC, the relationship between the expression level of CASP8 protein and clinicopathological characteristics was investigated. The results showed that CASP8 protein expression was found to be considerably correlated with the degree of differentiation and lymph node metastasis and that it was higher in ESCA cancer tissues than in nearby normal tissues. These findings demonstrated that CASP8 may have a critical role in ESCA progression. At present, there are only a few studies on CASP8 in esophageal cancer. Mikiko Takikita et al. reported that CASP8 was detected in both cytoplasm and nucleus and was correlated with histological differentiation.^36^ Li‐Yan Xue et al. reported that CASP8 was underexpressed in ESCA by using immunohistochemistry.^37^ Moreover, a study reported by QianZhi Ni et al. revealed a low expression level of CASP8 mRNA in ESCA samples in comparison with the adjacent normal tissues, negatively associated with clinicopathological features of patients with ESCC and positively associated with patient outcome.^38^ Conversely, Guiying Sun et al. found that ESCA patients had greater expression levels of CASP8 compared to healthy controls.^39^ A case‐cohort study in Japan revealed that the increased plasma CASP8 levels were significantly associated with ESCA risk.^40^ At present, the expression of CASP8 in esophageal cancer remains elusive and needs extensive studies.

The development of tumors is closely associated with both malignant cells as well as tumor immune microenvironment.^41^ Improving anticancer and immunotherapeutic effects is significantly enhanced by tumor‐infiltrating immune cells in the tumor microenvironment. Furthermore, the obtained results suggested that CASP8 expression was positively correlated with macrophages, CD8^+^ T cells, and B cells. CASP8 expression was negatively correlated with dendritic cells and uncorrelated with tumor purity, CD4^+^ T cells, and neutrophils. This result proved that, to a certain degree, CASP8 mRNA levels could reflect lymphocyte infiltration in ESCA, although we did not identify the cell type of infiltrating lymphocytes. To further explore the possible role of CASP8 in multiple immune cell infiltration in ESCA, we performed the relationship between CASP8 and several immune marker sets. In this study, we found that CASP8 was positively associated with PD‐1. PD‐1 and CTLA‐4 are markers of T cell exhaustion which is the key respect of immune escape that is the main reason for tumorigenesis. According to a reported study by Zou et al., Casp8 promotes the degradation of PD‐L1 by increasing the expression of A20. They further reported that the expression of Casp8 is a possible marker for detecting the response of anti‐PD‐L1/PD‐1 immunotherapy.^42^ Therefore, it may be possible to inhibit CASP8 expression to enhance T‐cell activation and suppress tumor formation. This study speculates that this is one of the reasons why low CASP8 expression is associated with a better prognosis for patients.

Cancer is characterized by the inability of the cells to undergo apoptosis.^43^ CASP8 somatic mutations have been identified in several types of cancers including colorectal cancer, gastric cancer, and head and neck cancer.^44^, ^45^, ^46^ Moreover, some studies have demonstrated that CASP8 genetic variants are associated with the occurrence and progression of ESCA,^47^ which also pertains to the Chinese population.^48^ Recently, whole genome sequencing of 508 pairs of ESCA tumors and matched adjacent normal tissues resulted in the identification of 22 significantly mutated genes, including CASP8 (2.95%).^49^ This percentage is approximately equal to the mutation rate of 2.6% that we found in esophageal cancer (Figure 4). Survival rates were found to be considerably varied between the unaltered and altered groups. In this view, the current study hypothesized that the CASP8 mutation could inhibit cell death and delay disease progression in the altered group. However, these claims remain unverified.

Examining the co‐expressed genes of CASP8 revealed that LY75 (CD205/DEC‐205) was matched by all three databases. Moreover, LY75 is expressed by B cells, T cells, NK cells, and monocytes as a type‐I transmembrane protein.^50^, ^51^ Hence, LY75 could be a candidate prognostic biomarker in melanoma patients.^52^ Consequently, this study hypothesized that CASP8 can likewise be used as a prognostic marker for ESCA. GO function and KEGG pathway analysis of the DEG of CASP8 suggested that the TNF signaling pathway, necroptosis, NF‐kappa B signaling pathway, apoptosis, and NOD‐like receptor signaling pathway, etc., were involved. The underlined signaling cascades affect the occurrence and development of ESCA either directly or indirectly.

Taken together, this study analyzed the expression level of CASP8 mRNA in the diagnosis and prognosis of ESCA patients using the application of bioinformatics methods and a combination of experiments. Here, we report a study supporting the role of CASP8 in ESCA. CASP8 mRNA expression was significantly higher in ESCA compared with adjacent normal tissues. CASP8 mRNA levels were correlated with the cancer stages, patient weight, tumor histology and nodal metastasis status of ESCA patients. As far as we know, this is the first study to report a consistent association between increasing CASP8 mRNA levels and poor prognosis in ESCA patients. In addition, the correlation between CASP8 and immune infiltration by analyzing, we found that there is a close relationship between CASP8 and immune infiltration, which provides theoretical guidance for subsequent clinical treatment, for example developing combined target therapy strategies. We must acknowledge that there are certain limitations to this study because the results were derived from data collected from publicly available databases. The association between CASP8 and prognosis may alter with the database updates. However, when more resources are collected, data stratification will become more precise and the results may become more reliable. Furthermore, the CASP8 expression was confirmed in only a small number of samples by using qPCR, WB, and IHC analyses. To ensure the accuracy of the results and to ascertain the prognostic function of CASP8 in ESCA, the sample size needs to be increased. Additionally, the specific mechanism of CASP8 in ESCA needs extensive studies.

## 5 CONCLUSIONS

Our findings reveal that CASP8 overexpression is an adverse prognostic factor in ESCA. CASP8 is positively associated with PD‐1 which is the key respect of immune escape that is the main reason for tumorigenesis. It may be possible to inhibit CASP8 expression to enhance T‐cell activation and suppress tumor formation. CASP8 expression is of great relevance to tumor‐infiltrating immune cells in the tumor microenvironment. CASP8 mRNA levels correlated with prognosis and immune infiltrating levels in ESCA, indicating that it can be used as a prognostic biomarker. The CASP8 altered group have a better prognosis and CASP8 mutation may inhibit cell death and delay disease progression. Together, our results offer new insight regarding the role of caspase‐8 in ESCA and its potential function as a regulator of tumor immunity.

## AUTHOR CONTRIBUTIONS

**jian chai:** Project administration (equal); writing – original draft (equal); writing – review and editing (equal). **yongqiang lei:** Data curation (equal); formal analysis (equal); methodology (equal); software (equal); validation (equal); visualization (equal). **xindong xiang:** Data curation (equal); investigation (equal); methodology (equal); validation (equal). **jing ye:** Investigation (equal); methodology (equal); resources (equal). **hang zhao:** Funding acquisition (equal); investigation (equal); resources (equal); supervision (equal). **lili yi:** Software (equal); writing – original draft (equal); writing – review and editing (equal).

## FUNDING INFORMATION

This research was supported by grants from Shandong Provincial Natural Science Foundation of China (grant No. ZR2017PH007) and Medical and Health Science and Technology Development Plan Project of Shandong Province (2016WS0214).

## CONFLICT OF INTEREST

The authors declare that they have no conflict of interest.

## Supporting information


Figure S1.

## Data Availability

The raw data supporting the conclusions of this article will be made available by the authors, without undue reservation.
