# Supplementary material for: High expression of caspase‐8 as a predictive factor of poor prognosis in patients with esophageal cancer
Source: Cancer Med. 2022 Dec 19;12(6):7651–66. doi: 10.1002/cam4.5496 (PMC10067063; doi:10.1002/cam4.5496)
Supplement: Supplementary file 1 — Figure S1. [file CAM4-12-7651-s001.docx]

**Supplementary information:**

**Normal1 Tumor1 Normal2 Tumor2 Normal3 Tumor3**


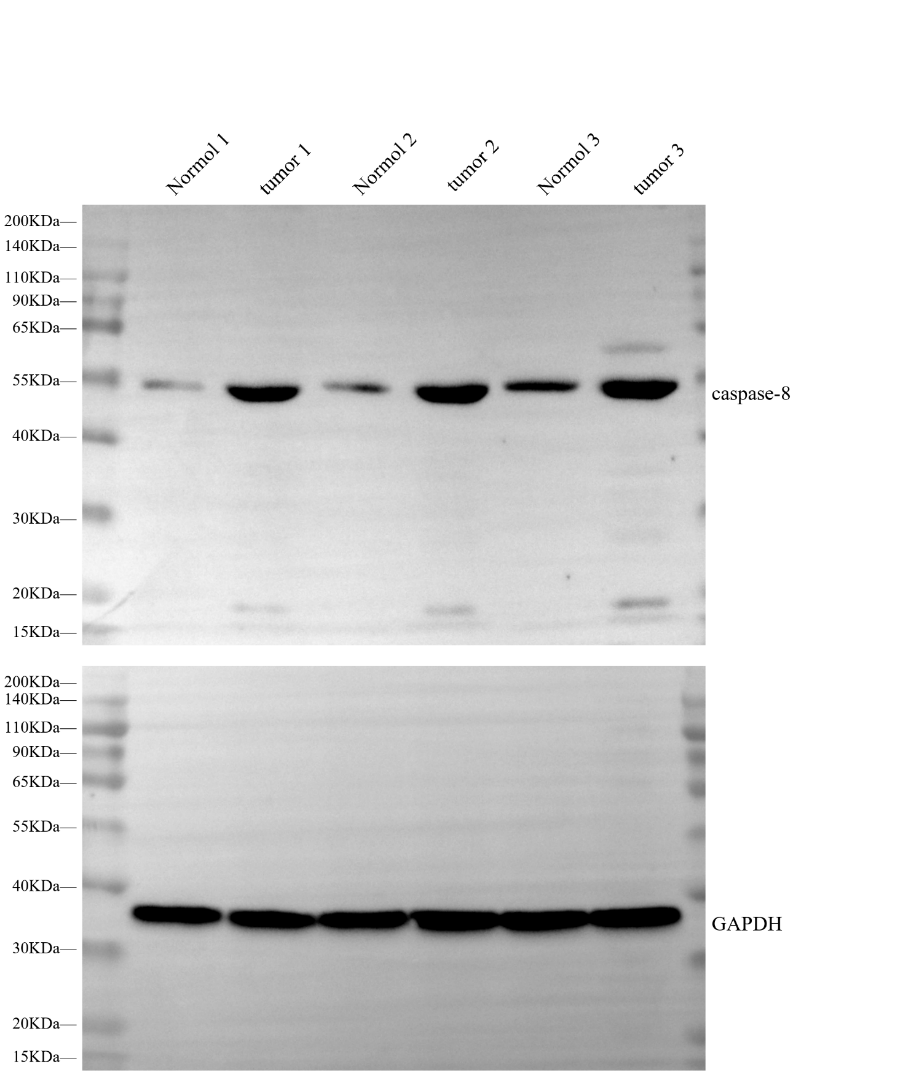


**Figure S1.** Western blot analysis revealed the presence of CASP8 in ESCC tumors and normal tissue.

Negative Low positive High positive

100×
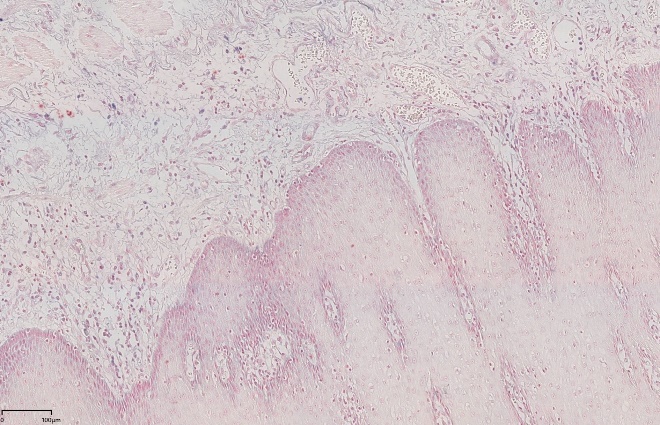

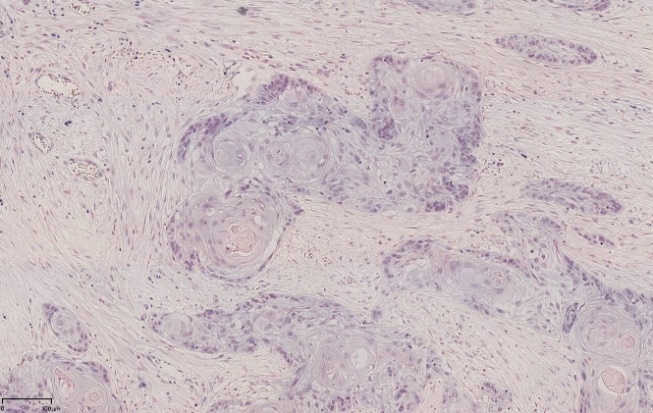

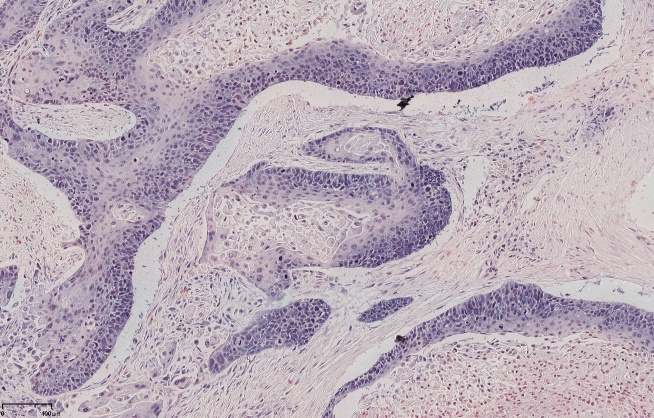


200×
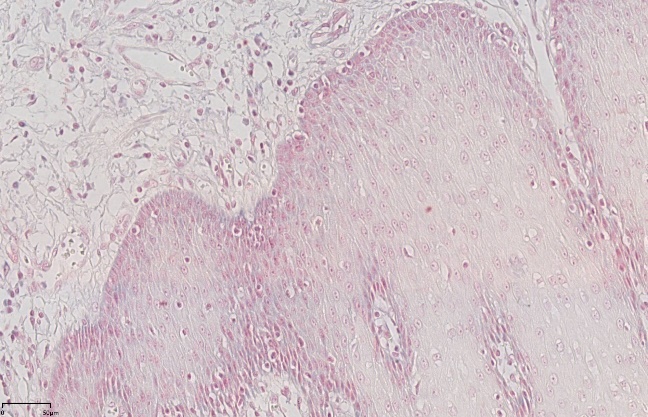

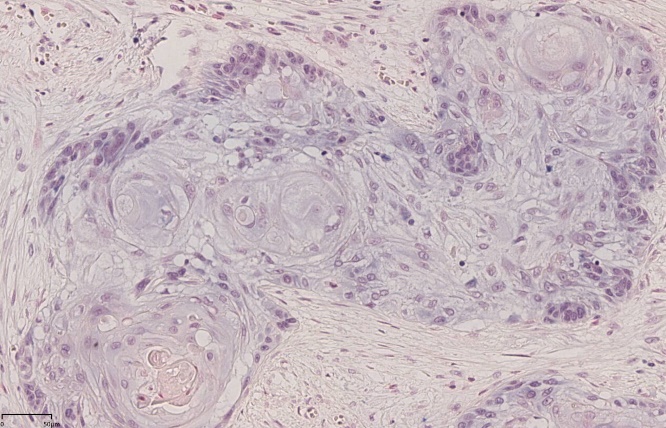

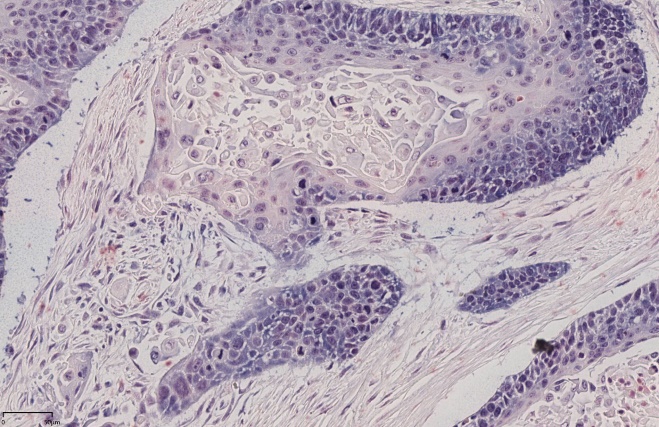


**Figure S2.** IHC assay results showed CASP8 expression. Herein, the 200x magnification was the lower, and 100x was the upper.
